# Interfacial ferroelectricity in marginally twisted 2D semiconductors

**DOI:** 10.1038/s41565-022-01072-w

**Published:** 2022-02-24

**Authors:** Astrid Weston, Eli G. Castanon, Vladimir Enaldiev, Fábio Ferreira, Shubhadeep Bhattacharjee, Shuigang Xu, Héctor Corte-León, Zefei Wu, Nicholas Clark, Alex Summerfield, Teruo Hashimoto, Yunze Gao, Wendong Wang, Matthew Hamer, Harriet Read, Laura Fumagalli, Andrey V. Kretinin, Sarah J. Haigh, Olga Kazakova, A. K. Geim, Vladimir I. Fal’ko, Roman Gorbachev

**Affiliations:** 1 Department of Physics and Astronomy, University of Manchester, Manchester, UK; 2 National Graphene Institute, University of Manchester, Manchester, UK; 3 National Physical Laboratory, Teddington, UK; 4 Kotel’nikov Institute of Radio-engineering and Electronics, Russian Academy of Sciences, Moscow, Russia; 5 Department of Materials, University of Manchester, Manchester, UK; 6 Henry Royce Institute for Advanced Materials, University of Manchester, Manchester, UK

**Keywords:** Electronic devices, Two-dimensional materials, Ferroelectrics and multiferroics, Surfaces, interfaces and thin films

## Abstract

Twisted heterostructures of two-dimensional crystals offer almost unlimited scope for the design of new metamaterials. Here we demonstrate a room temperature ferroelectric semiconductor that is assembled using mono- or few-layer MoS_2_. These van der Waals heterostructures feature broken inversion symmetry, which, together with the asymmetry of atomic arrangement at the interface of two 2D crystals, enables ferroelectric domains with alternating out-of-plane polarization arranged into a twist-controlled network. The last can be moved by applying out-of-plane electrical fields, as visualized in situ using channelling contrast electron microscopy. The observed interfacial charge transfer, movement of domain walls and their bending rigidity agree well with theoretical calculations. Furthermore, we demonstrate proof-of-principle field-effect transistors, where the channel resistance exhibits a pronounced hysteresis governed by pinning of ferroelectric domain walls. Our results show a potential avenue towards room temperature electronic and optoelectronic semiconductor devices with built-in ferroelectric memory functions.

## Abstract (web summary)

Marginal twisting of 2D semiconductor crystals enables the emergence of room temperature interfacial ferroelectricity.

## Main

Ferroelectrics are crystals that feature intrinsic charge polarization with two or more preferred stable directions of the polarization vector, determined by the lattice symmetry. Switching between those stable polarization states can be controlled by an external electric field, allowing various applications including non-volatile memory, microwave devices, transistors and sensors^1,2^. Of particular interest are ferroelectric semiconductors that would potentially allow field-effect transistors with additional functionality, for example storing information. However, it has proved challenging to find suitable materials that will both remain ferroelectric at room temperature and can be manufactured as the thin films required by the microelectronics industry^3^. The latter requirement is a severe challenge for traditional oxides due to their poor interface quality, which limits the realistically achievable homogenous ferroelectric layer thicknesses^1^. An alternative is to use layered crystals that can be cleaved or grown in ultrathin form while retaining surface quality. Only few such materials have been experimentally demonstrated so far, including in-plane ferroelectric SnTe (ref. ^4^), out-of-plane CuInP_2_S_6_ (ref. ^5^) and both types of ferroelectricity in different phases of In_2_Se_3_ (ref. ^6,7^). Up to now, out-of-plane switchable ferroelectricity at room temperature was achieved only in films thicker than 3 nm (ref. ^8^).

Recently, a new trend in creating truly two-dimensional (2D) ferroelectrics has emerged, which exploits interfacial charge transfer in stacked heterostructures of 2D materials. Layer-by-layer assembly of van der Waals heterostructures from various 2D crystals has matured into a sophisticated experimental field allowing for ultra-sharp and ultra-clean interfaces^9^ and control the rotation (that is, twist) angle between adjacent layers with high precision. Introducing twist gives rise to moiré superlattices: a periodic variation of a local atomic registry. For small twist angles (*θ* < 2°) 2D atomic lattices with similar unit cell sizes often undergo substantial reconstruction due to the energy gain from the preferential stacking domains balancing the cost of the resulting lattice strain^10,11^. Such commensurate domains have been recently shown to host ferroelectricity in marginally twisted wide band gap insulator, hexagonal boron nitride^12–14^ and in semimetallic WTe_2_ (ref. ^15^) with the ability to switch the domain type by application of an external electric field and sliding of atomic planes along the interface. Similar domain evolution has also been achieved by electrostatic gating of non-ferroelectric ABC/ABA trilayer graphene domains^16^.

Here, we report an observation of robust room temperature ferroelectricity in marginally twisted MoS_2_. While ferroelectricity in transition metal dichalcogenide (TMD) bilayers with parallel stacking of unit cells has been predicted theoretically^17^, and layer-polarized electronic band-edge states have been recently observed in electron tunnelling^10^ and optical properties^18^, the net charge transfer and, therefore, the steady-state electrical polarization has not been shown experimentally. We use a combination of electron channelling microscopy, Kelvin-probe force microscopy (KPFM) and electron transport measurements to explore ferroelectric domain networks in MoS_2_ bilayers and their dependence on the interlayer twist angle. We demonstrate that such domains can be switched by an external out-of-plane electric field and also find that domain switching produces a strong a response in the lateral electronic properties of the twisted MoS_2_ layers. Together with the extraordinary optical properties of TMDs^19^, our work offers a promising avenue towards designing new devices where both memory effect and optoelectronic functionality can be achieved within a single interface in the ultimate 2D limit.

## Electron imaging of domain networks and their response to transverse electric field

**Fig. 1:**
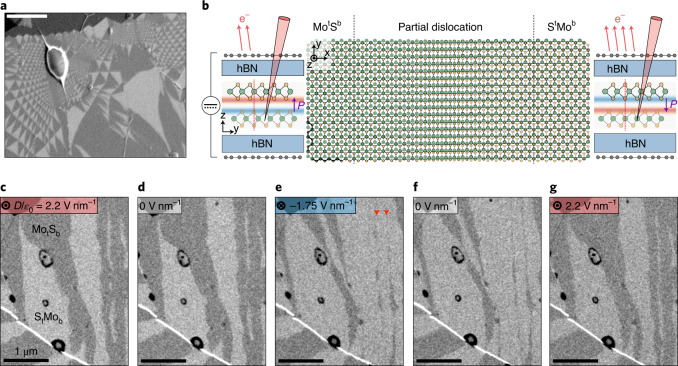
Ferroelectric domains in marginally twisted bilayer MoS_2_. **a**, Example of BSECCI acquired on unencapsulated twisted bilayer MoS_2_ placed onto a graphite substrate. Light and dark domain contrast corresponds to the two dominant stacking orders referred as Mo^t^S^b^ and S^t^Mo^b^. Scale bar, 1 μm. **b**, Centre: schematic demonstrating the transition from Mo^t^S^b^ to S^t^Mo^b^ with perfectly stacked bilayer regions separated by a partial dislocation. Side panels: the cross-sectional alignment of the MoS_2_ monolayers, viewed along the armchair direction, assembled within the double-gated device structure. Colour maps overlayed on top of the TMD atomic schematics show calculated charge density transferred between top and bottom layers, with red and blue corresponding to positive and negative charges, respectively. **c**–**g**, Domain switching visualized by BSECCI under different values of transverse electric field $D/\varepsilon _0$ applied in situ. Measurements have been performed on marginally twisted MoS_2_ encapsulated in hBN from both sides, placed on a graphite back gate and covered with graphene top gate as shown schematically in **b**. Large domains mostly retain their shape when the field is removed and practically disappear when the field is inverted; the arrows in **e** indicate partial dislocations colliding when neighbouring domains of the same orientation try to merge. Micrographs are presented in chronological order. The white oval feature in **a** and black ring features in **c**–**g** are where the intralayer contamination has segregated to form a bubble. Scale bars, 1 μm.

In homobilayers of MoS_2_, the period $\ell = a/\theta$ of the domain network is much longer than the lateral lattice constant, *a*, of MoS_2_ and is determined by the twist angle, *θ*. If the two MoS_2_ layers are oriented ‘parallel’, they form a bilayer of bulk 3R polytype when $\theta = 0^\circ ,$ and a triangular network of 3R commensurate domains when $0 < \theta < 2^\circ$. Such domains are separated by domain walls that have been identified as partial dislocations in earlier transmission electron microscopy studies^10^. To investigate the possible presence of ferroelectricity we have assembled bilayers of MoS_2_ using the tear and stamp technique^20^ aiming at a zero global misalignment angle. While ideally this could result in a 3R bilayer, small random deformations inflicted by the transfer process^21^ lead to a gradual variation of the misalignment angle $\left| \theta \right| < 0.1^\circ$. To visualize the resulting domain structure, we adopt back-scattered electron channelling contrast imaging (BSECCI), previously used for bulk materials^22^. We find that this technique provides a clear contrast even for twisted bilayers encapsulated under hexagonal boron nitride (hBN) crystals that were several nanometres thick, similar to the recently used channelling-modulated secondary electron imaging^23^ (for details, see Supplementary Information). An example of BSECCI in Fig. 1a shows triangular domains of varying sizes (from <100 nm to >1 μm), which enables us to establish and quantify the dependence of domain behaviour on their lateral dimensions. The stacking order of the two domain types (seen as regions of dark and light contrast in Fig. 1a) is illustrated schematically in Fig. 1b and denoted as Mo^t^S^b^ (S^t^Mo^b^) corresponding to the vertical alignment of molybdenum atomic positions in the top layer with sulfur positions in the bottom (and vice versa). As Mo^t^S^b^ can be seen as a mirror image of S^t^Mo^b^ stacking, they feature equal adhesion energies and therefore are expected to occupy similar areas of the sample, in agreement with our observations.

In contrast to TMD bilayers with 2H stacking (antiparallel) that possess both C_3_ rotational and inversion symmetry, the lattice of 3R bilayers is only C_3_-symmetric, having neither an inversion centre nor a mirror reflection plane. This asymmetry allows for a steady-state electrical polarization, which has been shown theoretically^17^ to result from an interlayer charge transfer due to asymmetric hybridization between the conduction band states in one (for example, top) layer and the valence band states in the other (for example, bottom) layer. Charge density transferred between the layers (red for positive and blue for negative charges), computed using density functional theory (implemented in the Quantum Espresso code^24^) and averaged over the MoS_2_ unit cell area, is shown in the side panels of Fig. 1b. This analysis indicates that the resulting double layer of charge resides on the inner sulfur sublayers (Fig. 1b), generating an areal density, *P* = ±3.8 × 10^−3^ *e* per nm, for the out-of-plane electric dipole moment pointing up/down in Mo^t^S^b^/S^t^Mo^b^ domains, respectively. This also agrees with the estimation, $P = {\it{\epsilon }}_0{\Delta}V^{{\mathrm{FE}}}$ obtained using the DFT-calculated ferroelectric (FE) potential of the stacking-dependent double layer, ${\Delta}V^{{\mathrm{FE}}}$ ($|{\Delta}V^{{\mathrm{FE}}}| = 63\,{{{\mathrm{mV}}}}$ for 3R stacking), described in the Supplementary Information. Coupling to this electrical polarization with an applied electric field favours—depending on the direction of the external field—either Mo^t^S^b^ or S^t^Mo^b^ stacking, providing a means to modify the domain structure.

To observe this ferroelectricity experimentally we encapsulated twisted MoS_2_ bilayers in hBN and used graphene on both sides to enable electrostatic gating (see Supplementary Information for further details). This design allowed us to apply a transverse field across the twisted bilayer without introducing any noticeable carrier density. First, we consider the effect of an applied field on a double-gated bilayer region containing elongated stripe domains as shown in Fig. 1c–g. These images show that the domain structure strongly varies with the application and reversal of the out-of-plane electric displacement, $D = {\it{\epsilon }}_0V{\it{\epsilon }}_r/h$ (where ${\it{\epsilon }}_r = 3.5$ is the relative permittivity of hBN^25,26^ and *h* is the total thickness between the graphene gates in nm), achieved by varying gate voltages, *V*. The domain configuration, prepared by applying ${\it{\epsilon }}_0^{ - 1}D = 2.2\,{{{\mathrm{V}}}}{{{\mathrm{nm}}^{-1}}}$ (the field pointing up), stays the same on removing the gate voltage (*D* = 0) (Fig. 1c,d). Then, the application of ${\it{\epsilon }}_0^{ - 1}D = - 1.75$ V nm^−1^ (the field pointing down) gradually expands the area of the lighter contrast domain type at the expense of the darker contrast ones. Again, if the gate voltage is swept back, the domain structure remains the same up to *D* = 0 and through a small interval of positive voltages, but then it returns to an almost identical configuration as was observed at the beginning of the hysteresis cycle (Fig. 1c,g). This behaviour allows us to assign Mo^t^S^b^ domain (polarization vector pointing up) to the darker contrast domains and vice versa. A more detailed study of the domain evolution on sweeping *D* is shown in Supplementary Fig. 2. Although the ‘darker contrast’ domains in Fig. 1e appear to be ‘squeezed’ to almost unnoticeable width (marked by red arrows), they still remain visible as thin line defects within the expanded light contrast domains. On reversal of the field, these lines serve as precursors for growing domains of opposite polarization (dark contrast in Fig. 1g). This behaviour indicates that a pair of partial dislocations at Mo^t^S^b^/S^t^Mo^b^ and S^t^Mo^b^/Mo^t^S^b^ boundaries combines into a topologically protected defect, a perfect dislocation between two domains of the same orientation (barely visible at the spatial resolution of Fig. 1c–g; see also below). Note that some domains change relatively little with gate voltage (see, for example, the bottom-left domain in Fig. 1c–g), which can be attributed to domain wall pinning by structural imperfections (sample edges, hydrocarbon bubbles and so on). Although the BSECCI imaging carries information only about the atomic structure of the observed domains, the hysteretic switching observed in response to an external electric field is a distinctive characteristic of ferroelectric materials^1^.

**Fig. 2:**
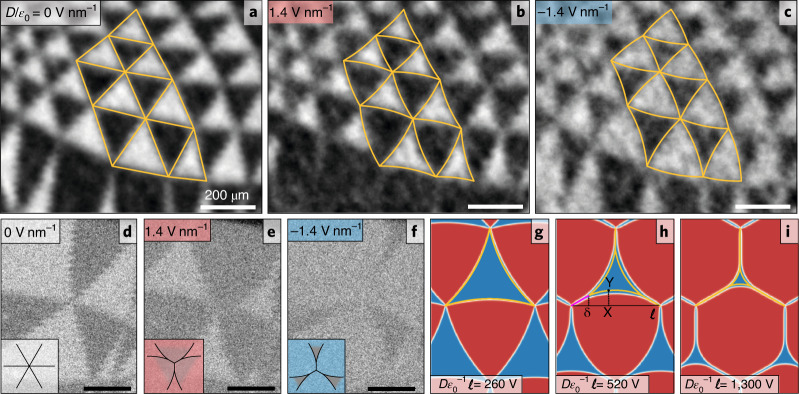
Domain evolution in double-gated marginally twisted MoS_2_ bilayers. **a**–**c**, BSECCI image of a triangular network of small domains undergoes expansion/contraction as a function of applied electric field ($D/\varepsilon _0 = 0\; {{\mathrm{Vnm}}^{-1}}\;({\mathrm{a}}), 1.4\; {{\mathrm{Vnm}}^{-1}}\;({{\mathrm{b}}}), -1.4\; {{\mathrm{Vnm}}^{-1}}\;({\mathrm{c}})$) overlaid with the analytical model equations (2) and (3), yellow lines. Scale bars, 200 nm. **d**–**f**, BSECCI image of larger domains, where the partial dislocations that constitute the domain walls merge near the nodes and the energetically disadvantaged domain collapses locally into a PSD at the applied electric field values of 1.4 Vnm^−1^ (e) and −1.4 V nm^−1^ (f). Micrographs are presented in their chronological order and the contrast is seen to deteriorate due to beam-induced surface contamination. Scale bars are 200 nm. **g**–**i**, Polarization maps for different values of scaling parameter computed using mesoscale relaxation of the bilayer lattice (Supplementary Information) and compared with the analytical model (yellow curves) of the scaled domain evolution given by equations (2) and (3). As the domain walls consist of two partial dislocations with Burgers vectors $\frac{a}{{\sqrt 3 }}\left( {1,0} \right)$ and $\frac{a}{{\sqrt 3 }}\left( {\frac{1}{2},\frac{{\sqrt 3 }}{2}} \right)$, line defects observed can be assigned to a PSD with Burgers vector $a\left( {\frac{{\sqrt 3 }}{2},\frac{1}{2}} \right)$.

A very different behaviour is observed in the areas hosting triangular domain networks on application of an electric field (Fig. 2a–c). Similar to Fig. 1c–g, the positive field favours the darker contrast domains and the negative field favours the lighter contrast ones. However, in these triangular networks, the nodes, where three domain walls intersect, remain fixed for all electric displacements. The domains expand (contract) by concave (convex) curvature of the domain walls, with the degree of curvature changing continuously with the applied electric field. Rounding of the walls starts as soon as the electric field is applied, without a discernible threshold in electric displacement *D*, and the effect is more pronounced for long domain walls. Characterizing domain walls by the minimum distance between the nodes (length, $\ell ,$ illustrated schematically Fig. 2h) we find that for relatively large domains ($\ell$ roughly 400 nm) the walls can be seen to merge (Fig. 2d–f). This begins where the partial dislocations are closest, near the nodes, and causes the triangular domains to shrink to less than 50% of the original size leaving a perfect screw dislocation (PSD) that connects the smaller triangular domain to the three surrounding nodes.

## Analytical description of domain wall behaviour

We describe the domain wall bending as a transverse displacement, $y\left( {0 < x < \ell } \right)$, of a single domain walls segment from a straight line ($y\left( x \right) = 0$) connecting two nodes. We analyse the variation of energy per supercell of an ideally periodic network (like in Fig. 2g–i), caused by the application of an out-of-plane electric field,

1 $$\begin{array}{lll}{{{\mathcal{E}}}}_\ell [y\left( x \right)] &\approx & 3\int \limits_\delta ^{\ell - \delta } \left[ {\left( {\frac{1}{2}\bar w + \tilde w} \right)y^{\prime 2} - 2\frac{{DP}}{{{\it{\epsilon }}_0\chi }}y} \right]{\mathrm{d}}x\\ & & +\, 6\left[ {\frac{u}{{\sqrt 3 }} - \bar w - \frac{{{{\mathrm{{\Omega}}}}}}{{\sqrt 3 }}\delta } \right]\delta .\end{array}$$

The first term in the integral part of ${{{\mathcal{E}}}}_\ell$ is related to the elongation of the partial dislocations accounted as $\sqrt {1 + y^{\prime 2}} - 1 \approx \frac{1}{2}y^{\prime 2}$, and their energy dependence, $\bar w + \tilde w\sin ^2\phi \approx \bar w + \tilde wy^{\prime 2}$, on the deviation angle, $\phi = \arctan y\prime \approx y\prime \equiv \frac{{{\mathrm{d}}y}}{{{\mathrm{d}}x}}$, of the domain wall axis from the closest armchair direction. In this parametrization, we can use the earlier-computed^27^ values of $\bar w =$1.05 eV nm^−1^ for the energy density of a partial dislocation aligned along the armchair direction, and $\tilde w =$ 0.68 eV nm^−1^ for its orientation-dependent part. This approximation assumes that $y^{\prime 2} \ll 1$ (justified in Supplementary Information). The second term in the integral accounts for the energy gain from the redistribution of the domain area, promoted by the displacement field *D*. Here, the coupling between the external field and the ferroelectric polarization density, *P*, is described using an effective dielectric screening parameter, *χ*, which we later estimate on the basis of the comparison with the experimentally observed evolution of domains with various sizes, $\ell$.

The remaining terms in equation (1) account for the possible merger of two partial dislocations into a PSD. The PSDs will separate two equivalently polarized domains near the network nodes and will be aligned along the MoS_2_ zigzag directions (at ±30^o^ from the armchair directions, which are also the partial dislocation orientations in the unperturbed domain network). Formation of a PSD requires a sufficiently high displacement field characterized by a threshold value, $D_ \ast (\ell ) \propto \ell ^{ - 1}$. The PSDs, are characterized^27^ by energy density, *u* = 2.24 eV nm^−1^, and, when projected onto the intervals $0 < x < \delta$ and $\ell - \delta < x < \ell$, set boundary conditions for the remaining partial dislocation segment, as $y(\delta ) = y(\ell - \delta ) = \delta /\sqrt 3$.

For $D < D_ \ast (\ell )$, the border between S^t^Mo^b^ and Mo^t^S^b^ domains is obtained by the minimization of energy in equation (1) and has a parabolic shape:

2 $$y(x) = \frac{{DP/\chi {\it{\epsilon }}_0}}{{\overline {{{{w}}}} + 2\widetilde {{{{w}}}}}}(\ell - x)x.$$

By fitting the experimentally observed evolution of domain shapes for various $\ell$ in Fig. 2a–c with equation (2), we estimate that the screening parameter is $\chi \approx 1.5$. This value enables us to establish the domain-length-scale dependent threshold,

3 $${\it{\epsilon }}_0^{ - 1}D_ \ast (\ell ) \approx \frac{{400\,V}}{\ell },$$

at which pairs of partial dislocations start merging near the network nodes into more energetically favourable PSDs. The merger occurs when the external electric field drives the partial dislocation in the neighbouring domains to touch each other. For more details, see Supplementary Information. For domains with $\ell$ roughly equal to 400 nm, this critical regime can be reached within a realistic range of electric fields, as observed experimentally in Fig. 2d–f. Theoretically, we estimate that, for $D > D_ \ast (\ell )$, the PSD segments grow in length near each node, as $\frac{\ell }{{\sqrt 3 }}\frac{{D - D_ \ast }}{D}$, whereas shorter partial dislocation segments retain an approximately parabolic shape (for comparison with the exact numerical solution, see Supplementary Fig. 11c). Figure 2g–i illustrates such an evolution, with the analytical results of equations (2) and (3) laid over a polarization map of a domain structure computed using mesoscale lattice relaxation (Supplementary Information).

## Surface potential studies using KPFM

Having established the inverted ferroelectric polarization inside Mo^t^S^b^ and Mo^b^S^t^ domains, we have also measured the potential, Δ*V*, created at the bilayer interface. To this end, we use two-pass phase-modulated-KPFM (PM-KPFM) on a non-encapsulated marginally twisted bilayer MoS_2_ (Supplementary Information).

**Fig. 3:**
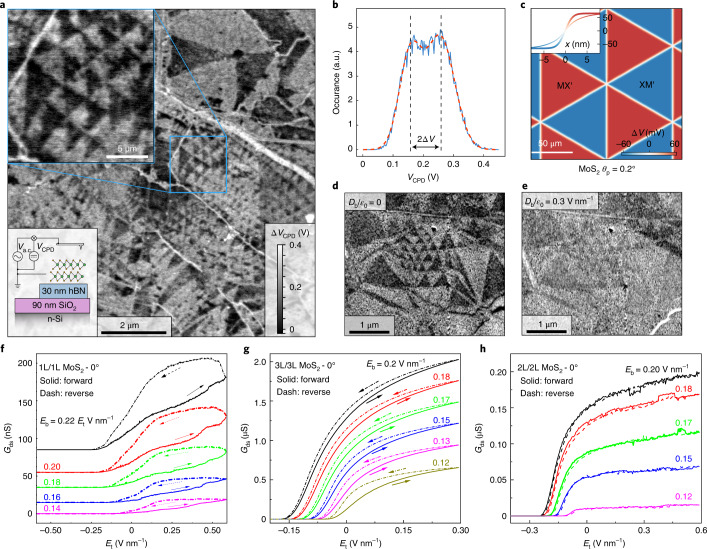
Electronic properties of ferroelectric domains in MoS_2_. **a**, Two-pass PM-KPFM map of the surface potential acquired with *V*_a.c._ = 5.75 V, and a time-average probe-sample distance of 37 nm during the second pass. The blue boxed inset magnifies the area used to extract numerical values of 2Δ*V*. The lower left inset shows the schematic of PM-KPFM measurement set-up. Scale bars 0.5 μm (upper) and 2 μm (lower). **b**, Typical histogram analysis used to extract surface potential difference. In this case, the data from the blue boxed area in **a** were used; for details and analysis of other areas see Supplementary Information. a.u., arbitrary units. **c**, Calculated surface potential distribution for experimentally relevant domain sizes. Inset shows the potential drop across the domain walls calculated considering (narrow line) and ignoring (bold line) piezoelectric charges. Scale bar, 50 nm. **d**,**e**, PM-KPFM surface potential map of a sample with no gate voltage (**d**) and with back-gate voltage applied indicating disappearance of the potential variation when free electrons are introduced (**e**). Scale bars, 1 μm. **f**,**g**, Hysteretic behaviour of electrical conductivity *G*_sd_ of our artificially made ferroelectric semiconductors as a function of top-gate electric field (*E*_t_) for different back-gate electric fields (*E*_b_). The shown curves are for 1L MoS_2_ on top of 1L MoS_2_ at 350 K (**f**) and 3L/3L MoS_2_ at room temperature (**g**) twisted by 0° to achieve the 3R interface. We used the top gate for recording hysteresis because it covers only the twisted region whereas the bottom gate influences a much larger area, including contact regions. **h**, Similar measurements on a reference 2L/2L sample twisted by 0°, which produces a 2H interface and displays much smaller hysteresis with the opposite sign (room temperature).

The resulting map of the surface potential acquired is shown in Fig. 3a, where the two types of domain display a clear contrast difference. This difference, measured with a conductive scanning probe several nanometres above the sample’s surface, indicates the presence of a transverse electric field built-in to the domains polarized in the opposite directions for Mo^t^S^b^ and Mo^b^S^t^ regions, respectively. The potential gained due to such fields with respect to the metallic n-Si back gate, ±Δ*V*, is measured experimentally as a 2Δ*V* jump when a boundary between two domains is crossed. A histogram of the local potential, Fig. 3b, displays two major peaks corresponding to the dark and light contrast domains with the separation of 2Δ*V* = 100 ± 20 mV, in close quantitative agreement with the theoretically predicted^17^ value, 2Δ*V* = 126 mV, and the potential map in Fig. 3c. The measured Δ*V* was found to be reproducible for various locations on the sample and also consistent with the results obtained for another sample placed on graphite substrate (Supplementary Information). On applying a positive back-gate voltage to the sample, we observed that the domain pattern gradually vanishes, as expected due to screening of the ferroelectric field by the free electrons. For electron densities of roughly 10^13^ cm^−2^ the pattern becomes barely visible (Fig. 3d,e), however, the domain shape changes very little since the electric field inside the bilayer is substantially screened by the bottom MoS_2_ layer in the case of one-sided gating. On application of the negative gate voltage no change in contrast is observed (Supplementary Fig. 9) as no mobile holes are induced in our samples. Note that the piezo-charges reported earlier^10^ could partially compensate for the ferroelectric charge transfer. However, piezoelectricity has little effect on the potential in the middle of large domains, as it is caused by strain localized within several nanometres of the domain walls (see the narrow and bold curves in the inset of Fig. 2c), which is below our lateral resolution.

## Ferroelectric response in the conductivity of field-effect devices

For a 2D ferroelectric semiconductor, the potential difference between the oppositely polarized domains should translate into gate-controlled doping and, therefore, electron transport through such ferroelectric field-effect transistor devices should depend on the ferroelectric domain distribution. To observe this, we have prepared and studied several heterostructures consisting of marginally twisted pairs of mono-, bi- and tri-layers (L) of MoS_2_. Parallel alignment for twisted monolayer-on-monolayer and trilayer-on-trilayer devices produces ferroelectric 3R interfaces between the twisted layers. However, for 2L this alignment leads to predominantly centrosymmetric 2H stacking, which has no electric polarization. These heterostructures were encapsulated in hBN and double gated (the top gate was deposited only above the main channel, whereas the bottom gate (Si wafer) was global). At zero gate voltages, all the devices were found to be insulating, and required an applied electric field of roughly 0.1 V nm^−1^ (that is, *n* ≅ 2 × 10^12^ cm^−2^) to start inducing mobile carriers. Therefore, to study the conductivity, *G*_sd_, we applied a finite positive bottom gate voltage *V*_b_, which was essential to induce electrical conductance in the contact regions, and then swept the top-gate voltage *V*_t_. Because of the generally low carrier mobility of MoS_2_ channels at room temperature, only two-probe measurements were possible, with source-drain resistances reaching above MΩ in magnitude. Figure 3f,g shows the hysteresis observed for twisted 1L and 3L MoS_2_ ($\theta = 0^\circ$). For further examples, see the Supplementary Information. Sweeping *V*_t_ controls both the carrier concentration and the out-of-plane electric field, which leads to redistribution between domains with opposite polarities, depending on whether positive or negative gate voltage is applied, as observed in our microscopy measurements in Fig. 1. Pinning of ferroelectric domain boundaries for up and down sweeps results in memory effects and hysteresis. As a result, the sample-averaged carrier densities for the same *V*_t_ for up and down sweeps differ, leading to the observed difference in conductivities seen in Fig. 3f,g. Our reference devices made from 2L MoS_2_ with predominant 2H stacking (Fig. 3h) and exfoliated trilayer of 2H-MoS_2_ (Supplementary Fig. 13b) showed a very similar electrical response to applied gate voltages but no discernible hysteresis, because such devices do not feature switchable ferroelectric interfaces. We also tested few-layer MoS_2_ devices with 3R stacking. The material is an intrinsic ferroelectric, but no switching or hysteresis was observed for the prepared devices for all accessible gate voltages (Supplementary Fig. 13c). This is attributed to much stronger pinning at the 3R device edges than for movements of domain walls in marginally twisted MoS_2_.

## Conclusions

To summarize, our study shows that switchable ferroelectric behaviour is a generic property of heterostructures assembled from atomically thin TMDs with small twist angles providing a 3R interface. Having demonstrated this by studying field-driven domain evolution in both structural (BSECCI) and electronic (KPFM and transport) properties, we have also quantified the amount of charge transfer in the ferroelectric double layer at the interface and found good agreement with theoretical modelling. These observations demonstrate a way towards atomically thin electronic devices with memory effects and open up possibilities for the design of new (opto)electronic devices. For example, strong light-matter coupling in TMDs^28^ and the single-photon emission by defects in individual TMD layers^29^ may offer a switchable single-photon emission capability. However, to realize such functional devices, further progress in van der Waals assembly is required to achieve higher interfacial homogeneity and reproducibility of twist angles. Additionally, materials parameters need to be optimized to achieve weaker domain pinning and faster switching.

## Methods

### Scanning electron microscopy

Samples were imaged with a Zeiss Merlin scanning electron microscope operated at 1.5 keV and probe current of 1.1 nA. The backscattered electron signal is collected at a 5 mm working distance and using an in-lens EsB detector with a 73° take-off angle and an applied energy selected grid bias of 500 V. The optimal tilt angle was established experimentally to be 20.1° while the azimuthal rotation was optimized for each sample before the measurements. For the optimized angular conditions, different acceleration voltages have been tested in the range of 1 to 6 kV, with optimal results achieved for 1.3–1.7 kV. We have found that domains can be clearly seen in BSECCI images, despite overlay of the multilayer hBN and graphene (see Supplementary Information for more details).

Two-pass PM-KPFM has been performed in high vacuum. First, the sample topography acquired in the tapping mode with the probe grounded. Then, the probe is lifted at a constant height and scanned following the sample’s topography, while an a.c. voltage, *V*_a.c._, is applied to the probe to create the oscillating electrostatic force that is further minimized by applying a d.c. voltage. This d.c. voltage is equal to the contact potential difference between the tip and the sample and allows us to extract the local work function of the surface for a known tip material. The KPFM measurements have been optimized against both lift height and *V*_a.c._ (Supplementary Information).

## Online content

Any methods, additional references, Nature Research reporting summaries, source data, extended data, supplementary information, acknowledgements, peer review information; details of author contributions and competing interests; and statements of data and code availability are available at 10.1038/s41565-022-01072-w.

## Supplementary information

Supplementary Information Supplementary information including several sections describing experimental details and providing supporting data.

## Data Availability

Additional data related to this paper are available from the corresponding authors on reasonable request.

## Code availability

The computer code used for the image filtering is available from the corresponding authors on reasonable request.
