# Supplementary material for: Interfacial ferroelectricity in marginally twisted 2D semiconductors
Source: Nat Nanotechnol. 2022 Feb 24;17(4):390–5. doi: 10.1038/s41565-022-01072-w (PMC9018412; doi:10.1038/s41565-022-01072-w)
Supplement: Supplementary file 1 — Supplementary information including several sections describing experimental details and providing supporting data. [file 41565_2022_1072_MOESM1_ESM.pdf]

nature portfolio

https://doi.org/10.1038/s41565-022-01072-w

Supplementary information

Interfacial ferroelectricity in marginally twisted 2D semiconductors

In the format provided by the authors and unedited

Supplementary information for

"Interfacial ferroelectricity in marginally twisted 2D semiconductors"

Astrid Weston, Eli G Castanon, Vladimir Enaldiev, Fabio Ferreira, Shubhadeep Bhattacharjee, Shuigang Xu, Héctor Corte-León, Zefei Wu, Nickolas Clark, Alex Summerfield, Teruo Hashimoto, Yunze Gao, Wendong Wang, Matthew Hamer, Harriet Read, Laura Fumagalli, Andrey V Kretinin, Sarah J. Haigh, Olga Kazakova, A. K. Geim, Vladimir I. Fal'ko, and Roman Gorbachev

Section 1: Scanning Electron Microscopy – sample fabrication

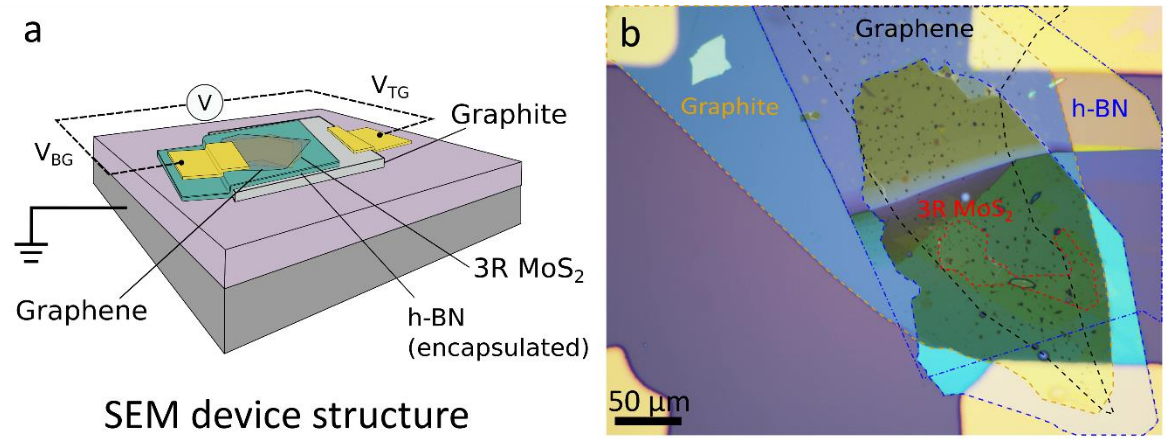

Figure S1: Samples for electron microscopy studies. Schematic of double-gated device structure used for BSEECCI measurements **(a)** and optical image of a typical sample **(b)**.

For the fabrication of the twisted 3R-stacking type MoS_2 bilayers we utilised a remotely controlled micromanipulation transfer rig housed inside an argon atmosphere. We employed a modified tear-and-stack technique [1] as previously used in our earlier work [2]. Monolayers of MoS_2 were mechanically exfoliated onto Si/SiO2 (90nm) coated with polypropylene carbonate (PPC). A poly-methyl methacrylate (PMMA) carrier layer was used to pick up one half of a MoS_2 flake and subsequently the second half with a rotation angle of $\theta \approx 0^o$. A graphite/h-BN heterostructure was prepared using a standard PMMA-based dry transfer technique, and the twisted MoS_2 bilayer was placed on top avoiding overlap with the graphite back-gate. Following this, a thin hBN and graphene layers have been deposited on top of the structure, again avoiding direct electrical contacts between the graphene top gate, MoS_2 twisted bilayer and graphite back-gate. We find that ECCI domain contrast decreases with the hBN encapsulation and therefore the top hBN was selected in the range

of 2-3 nm which allowed gating without tunnelling into the twisted bilayer. Typically, domain contrast is significantly reduced with a h-BN thickness above 5 nm regardless of the acceleration voltage used. The top graphene and bottom graphite electrode were contacted using Ti (3 nm)/ Au (40 nm) electrodes deposited though a shadow mask to minimise contamination.

Section 2: Extended sequence of BSE ECCI measurements.

Samples were imaged with a Zeiss (Germany) Merlin scanning electron microscope operated at 1.5 keV and probe current of 1.1 nA . Over 73 degrees of take-off angle of backscatter electrons are collected by in-lens EsB detector with applied energy selected grid bias of 500 V at 5 mm WD.

The optimal tilt angle was established experimentally to be 20.1° while the azimuthal rotation was optimised for each sample prior to the measurements. For the optimised angular conditions, different acceleration voltages have been tested in the range of 1 kV to 6 kV, with optimal results achieved for 1.3 – 1.7 kV.

The hBN-encapsulated devices have demonstrated lower domain contrast values as compared to exposed twisted MoS_2 bilayer. For instance, Fig.1a of the main text shows an example of BSECCI acquired on an exposed sample; versus Fig.1c-g which is a BSECCI series acquired on a device encapsulated with 2.4 nm hBN and a monolayer graphene. While the contrast was digitally enhanced to be the same for both images, the latter figure demonstrates noticeably lower signal-to-noise ratio even though the imaging conditions were almost identical. This signal-to-noise ratio necessitated acquisition time of few minutes per image for most micrographs, including those shown in Fig.1c-g and Fig.2a-f of the main text.

In addition, the prolonged imaging has led to surface hydrocarbon contamination build-up [3] and as a result the image quality deteriorates with each subsequent frame, as can be seen in Fig.2a-f. The problem is more pronounced for high magnification imaging and has been partially mitigated by cleaning the SEM chamber with plasma and extended pumping time prior to the imaging. However, we avoided plasma cleaning with the sample present in the SEM chamber due to potential damage to the exposed graphene top-gate.

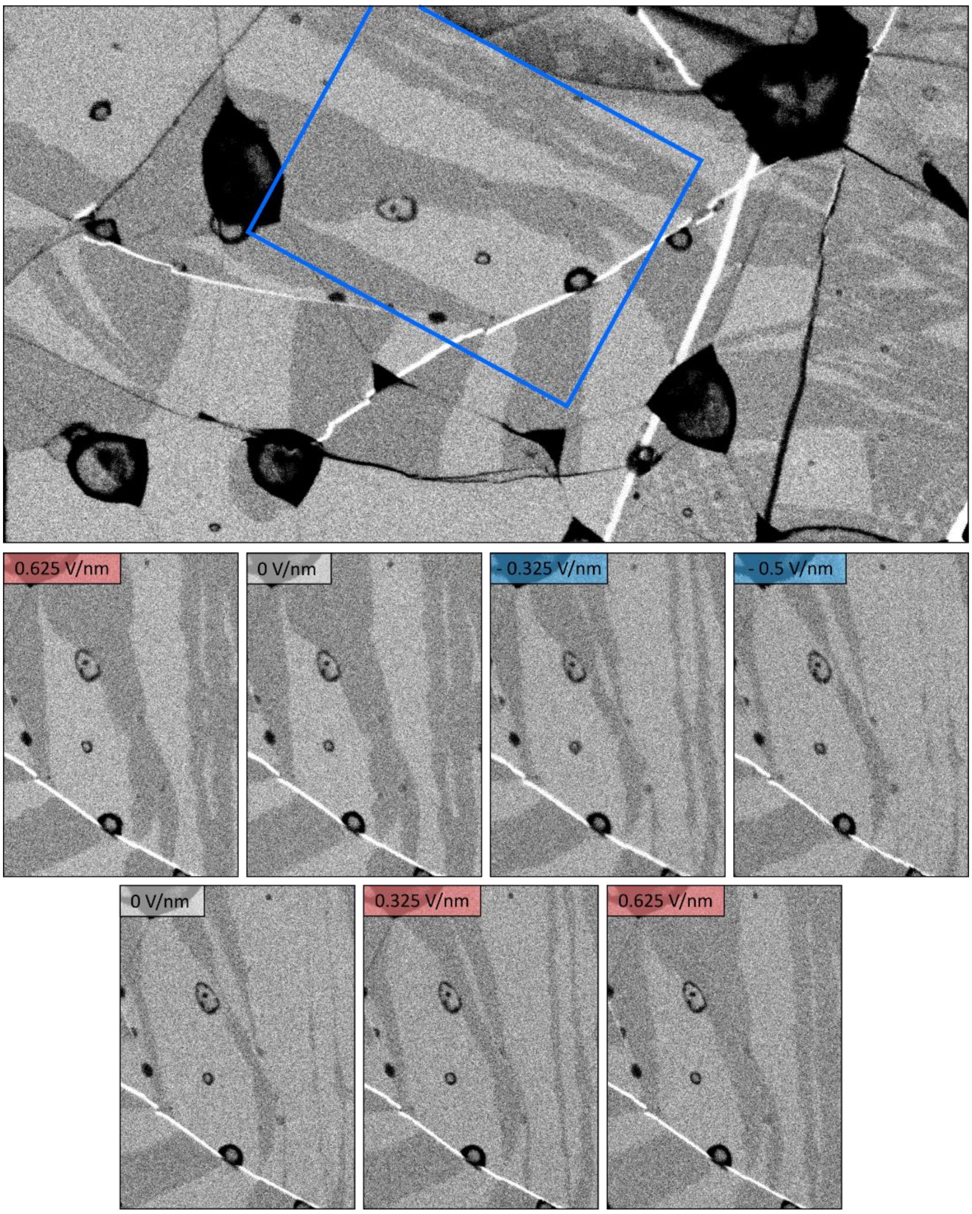

Figure S2: Extended data series for the sample described in Fig. 1 of the main text and Supplementary Section 1. Top panel – initial sample mapping. Further panels are focused on the region of interest marked by the blue box. Micrographs presented in chronological order and acquired using 1.5 kV beam energy, 1 nA beam current and in-lens back scattered electron detector. Sample tilt 20.2°.

Section 3: Kelvin probe force microscopy (KPFM) sample fabrication

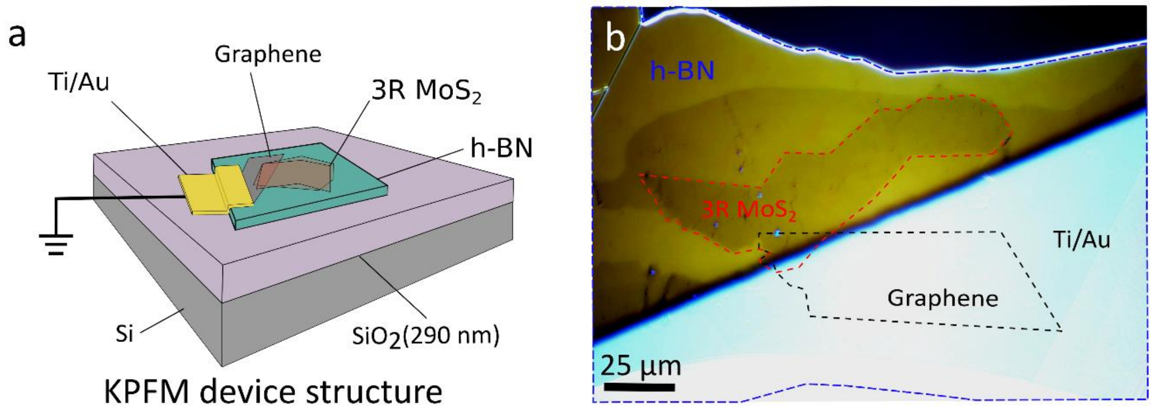

Figure S3: Samples for KPFM studies. (a) Schematic of the device structure placed on the hBN substrate and **(b)** optical micrograph of the sample described in the main text.

Samples for the KPFM studies have been fabricated following a similar strategy as described in Section 1. Here, the sample was not encapsulated to allow performing surface potential measurements. A graphene layer was used to produce a reliable contact between the MoS_2 and the top gold electrode and allow grounding of the twisted sample and/or controlling the potential of the sample for KPFM measurements. The metal contact was deposited through a copper TEM grid acting as the shadow mask, and a titanium (3nm)/gold (40nm) electrode was electron-beam evaporated to contact the graphene electrode/MoS_2 bilayer.

Prior to performing KPFM measurements, the samples were cleaned by annealing them in high vacuum at 200°C for 30 mins. The regions of interest were then brushed with an AFM probe in contact mode [4] to remove excess hydrocarbons.

Section 4 KPFM imgaing of 3R MoS_2 domains on hBN

Surface potential maps were acquired in vacuum ($\sim10^{-7}\mathrm{mbar}$) with an NTEGRA AURA system using highly doped Si probes (i.e. PFQNE-Al, Bruker with k~ 0.8 N/m) in a two-pass phase-modulated Kelvin-probe force microscopy (PM-KPFM) [5]. Such technique measures the surface potential (i.e. the contact potential difference or $\Delta\boldsymbol{V_{CPD}}$) of the sample by applying a compensation DC voltage to the tip that nullifies the electrostatic force between the probe and the sample. The feedback loop finds the voltage that nullifies the force using phase modulation, and it is thus sensitive to the gradient of the electrostatic force [5], providing high spatial resolution and repeatability [6]–[8]. For more details on the effect of varying parameters on the surface potential see refs [9]–[13].

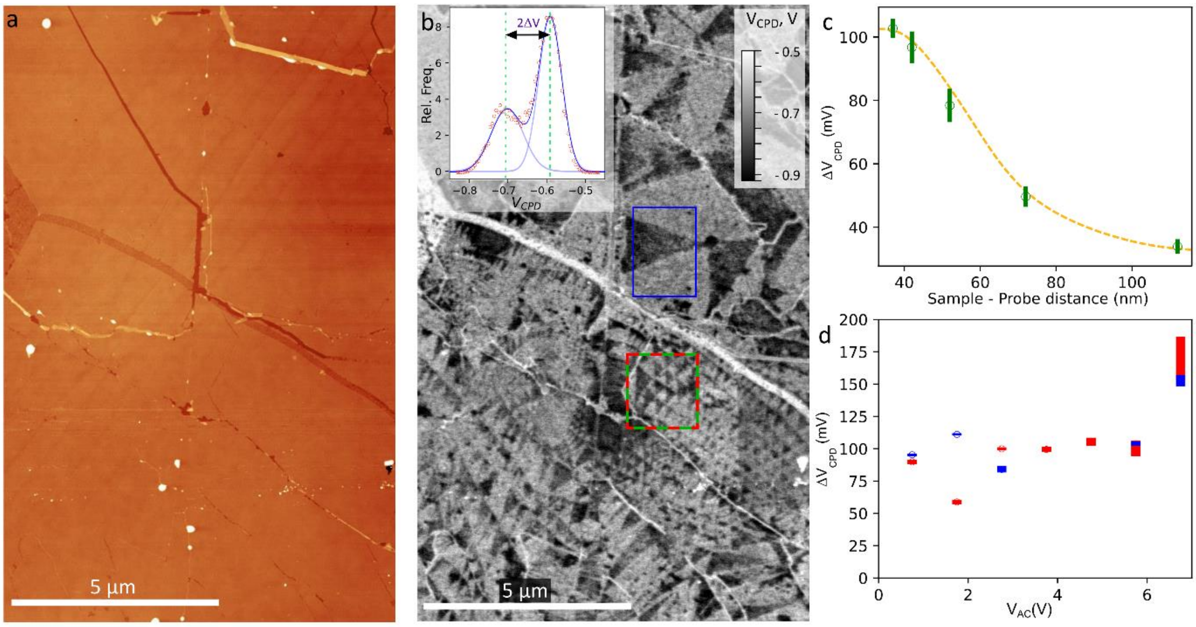

Figure S5: Tapping-mode topography image acquired in the first pass **(a)** and subsequent surface potential map acquired on the second pass **(b)** with an applied voltage $\boldsymbol{V_{AC}}$ = 5.75 V and distance to sample of 37 nm during the second pass. Inset shows a histogram with Gaussian fitting used to extract $\Delta\boldsymbol{V_{CPD}}$ as the difference between bright and dark domains. **(c)** $\Delta\boldsymbol{V_{CPD}}$ as a function of the distance to the surface taken from the region in the blue box. **(d)** $\Delta\boldsymbol{V_{CPD}}$ as a function of $\boldsymbol{V_{AC}}$ plotted for the regions highlighted in matching colours in (b).

In order to obtain consistent results across several samples and probes, probe-sample distance, and applied AC voltage parameters were optimized. Probe-sample distance was measured performing a Z-ramp, while monitoring the probe oscillation amplitude. From the obtained curve, the distance to the sample (~32 nm) and the free oscillation amplitude (~50 nm peak-to-peak) were extracted. The distance to sample during the first pass was taken as the distance between the amplitude during the first pass and the zero amplitude position (i.e. sample-probe in contact). The distance during the

second pass was taken as the distance during the first pass plus the lift height. The free amplitude was taken as the distance from the zero-amplitude position to where the amplitude stops increasing.

The ΔV_{CPD} dependence on the distance to the sample during the second pass was measured while applying a V_{AC} =5.75 V and f_{AC} ~2 kHz to the probe to determine the optimal distance to the sample for the second pass (as it will be shown below, V_{AC}=5.75 V is not the optimal value for the AC voltage to obtain quantitative values of surface potential, but it provides a good contrast to optimize the distance to the sample during the second pass). As illustrated in Supplementary Fig. 5c, the contact potential difference is flat at a distance to sample of 37-42 nm, thus a 37 nm distance was therefore utlised in the KPFM measurements. Note that this is an empirical approach to find the optimal parameters that asumes that close to the optimal configuration the surface potential tends to a fixed value. A more detailed analisis of the different sources of error in KPFM can be found in Refs. [14], [15].

Lastly, the ΔV_{CPD} was measured as a function of the applied AC voltage to the probe, V_{AC} with the probe-sample distance of 37 nm, in order to obtain the optimal value of the AC excitation of the probe, as shown in Fig. S5d, and in the detailed surface potential maps acquired over a smaller region of the sample in Fig. S6.

The optimisation of these two parameters allows the obtention of reliable values of the surface

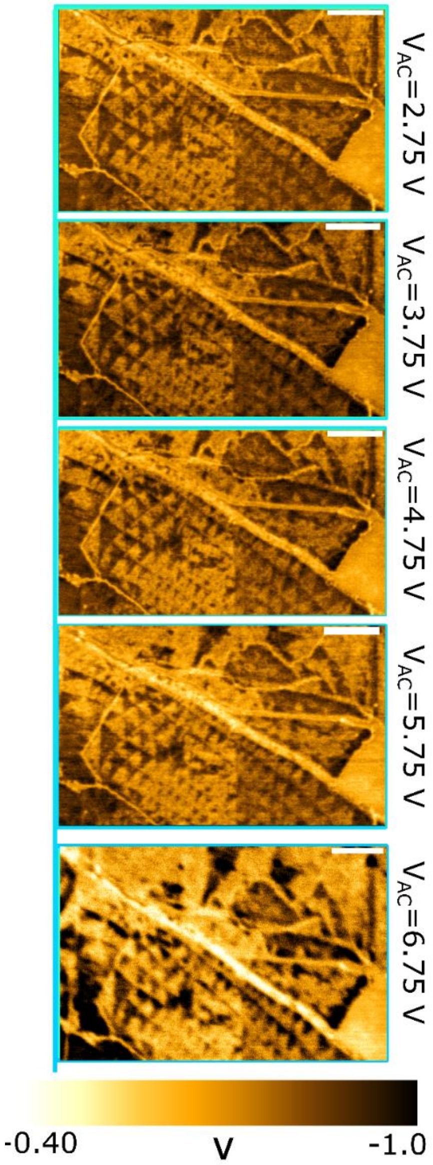

Figure S6: Surface potential detail map acquired on the second pass with the probe-sample distance of 37 nm for different V_{AC} applied to the probe. Scale bar: **1 μm**.

potential difference between the Mo^tS^b and S^tMo^b domains. At large V_{AC} the measured value ΔV_{CPD} is seen to increase due to electrostatic doping of MoS_2 which increases effective coupling with the tip, therefore small values $1\text{ V} < V_{AC} < 3\text{ V}$ have been used to extract correct ΔV_{CPD} values.

Section 5: KPFM imaging on 3R MoS_2 on graphite

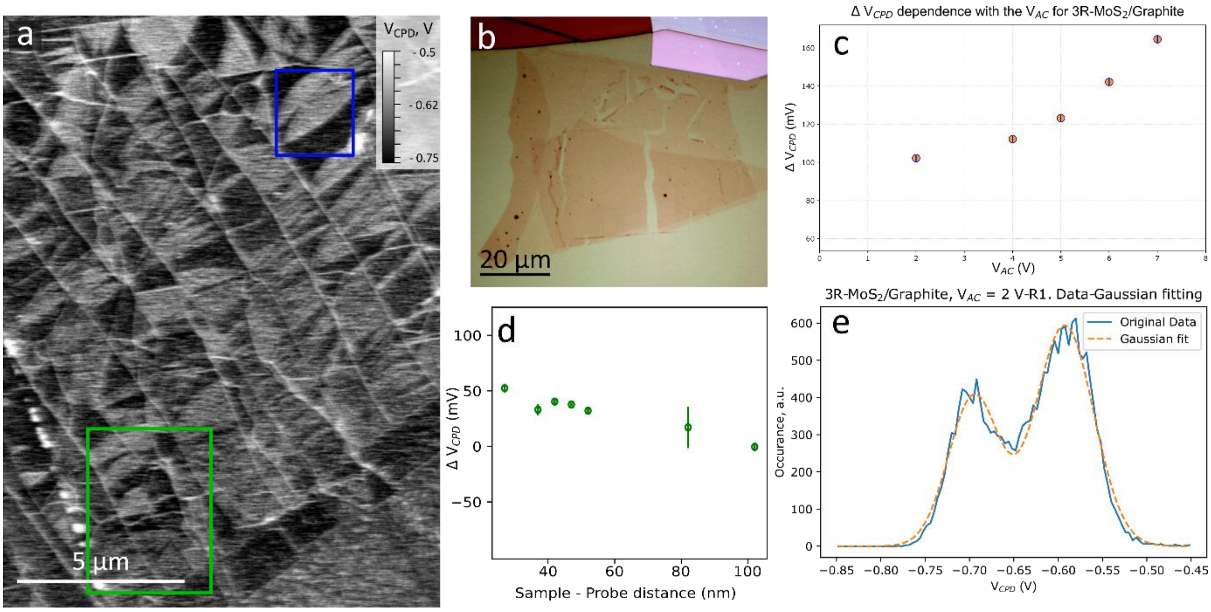

Figure S7: KPFM studies of MoS_2 on graphite: (a) Surface potential map obtained with an applied voltage $\boldsymbol{V_{AC}}$ = 2 V and the probe-sample distance of 37 nm. (b) Corresponding (zoomed out) optical micrograph of the sample. (c) Contact potential difference ($\Delta\boldsymbol{V_{CPD}}$) as a function of $\boldsymbol{V_{AC}}$ plotted for the blue boxed region highlighted in (a). (d) $\Delta\boldsymbol{V_{CPD}}$ as a function of the distance to the sample for the region in the blue box. (e) Histogram with Gaussian fitting highlighting the difference in contact potential difference between bright and dark domains (taken from blue boxed region).

KPFM measurements have also been performed on a samples of twisted MoS_2 bilayer placed on exfoliated graphite. The corresponding surface potential map obtained at a ΔV_{AC} = 2 V applied to the probe is shown in Fig. S7a, while the topography image is shown in Fig. S6b. As for the previous sample, we acquired the ΔV_{CPD} dependence on the ΔV_{AC} using the same distance to sample of ~ 37 nm, as shown in Fig S7c. The value of 100 mV obtained at low ΔV_{AC}= 2V is within the experimental accuracy of the value measured on hBN and predicted theoretically.

Whilst measuring ΔV_{CPD} at increasing values of ΔV_{AC}, domain wall sliding was observed between consecutive scans. In Fig. S8, domains of opposite contrast (outlined with blue and green dashed lines) have significantly altered in size and in shape. The blue and green arrows in the second and third image indicate the direction of the domain wall movement relative to the initial scan. Further domain wall sliding was observed when ΔV_{AC} was increased from 2.75 V to 6 V. However, we find that using a double-gated configuration leads to more reliable switching behaviour, since the tip can only affect a relatively small area of the sample.

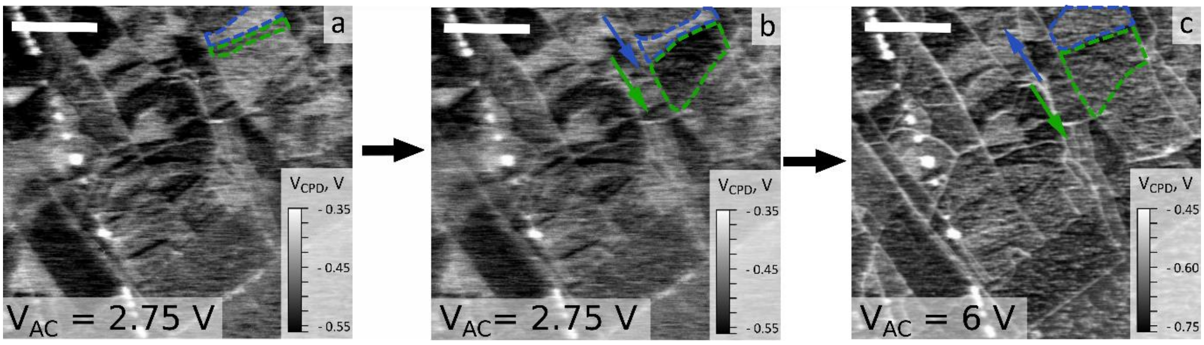

Figure S8: Domain switching using a biased KPFM tip. The surface potential maps measured at 0 nm lift height and V_{AC} = 2.75 V **(a)**, 5 nm lift height V_{AC} = 2.75 V **(b)**, and 5nm lift height V_{AC} = 6 V **(c)**.

KPFM was also used to study the effect of electrostatic doping on the ferroelectric behaviour. For this experiment, a twisted bilayer MoS_2 was placed on hBN/graphite stack (schematically shown in Figure S9(a)). To allow electrostatic gating, MoS_2 bilayer and graphite were contacted using shadow mask deposition of Cr/Au. Prior to KPFM measurements an I-V curve was taken to ensure integrity of the gate dielectric (Figure S9(a)). The KPFM measurements where performed using the parameters discussed previously (two-pass with probe grounded during the first pass, and V_{AC} = 1.25 V, f = 2 kHz, and distance to sample ~ 37 nm during the second pass). The results for several different backgate values can be seen in Figure S9 (b) to (f) where a gradual decay of the domain contrast is seen as the voltage is increased. For the hBN gate dielectric thickness of 30 nm we estimate electron density approaching 10^{13} cm^{-2} at 10 V, at which point the surface potential differnece between the two domain types becomes < 10 mV. No such contrast decay has been observed for negative gate voltages, Fig. S9(g-i).

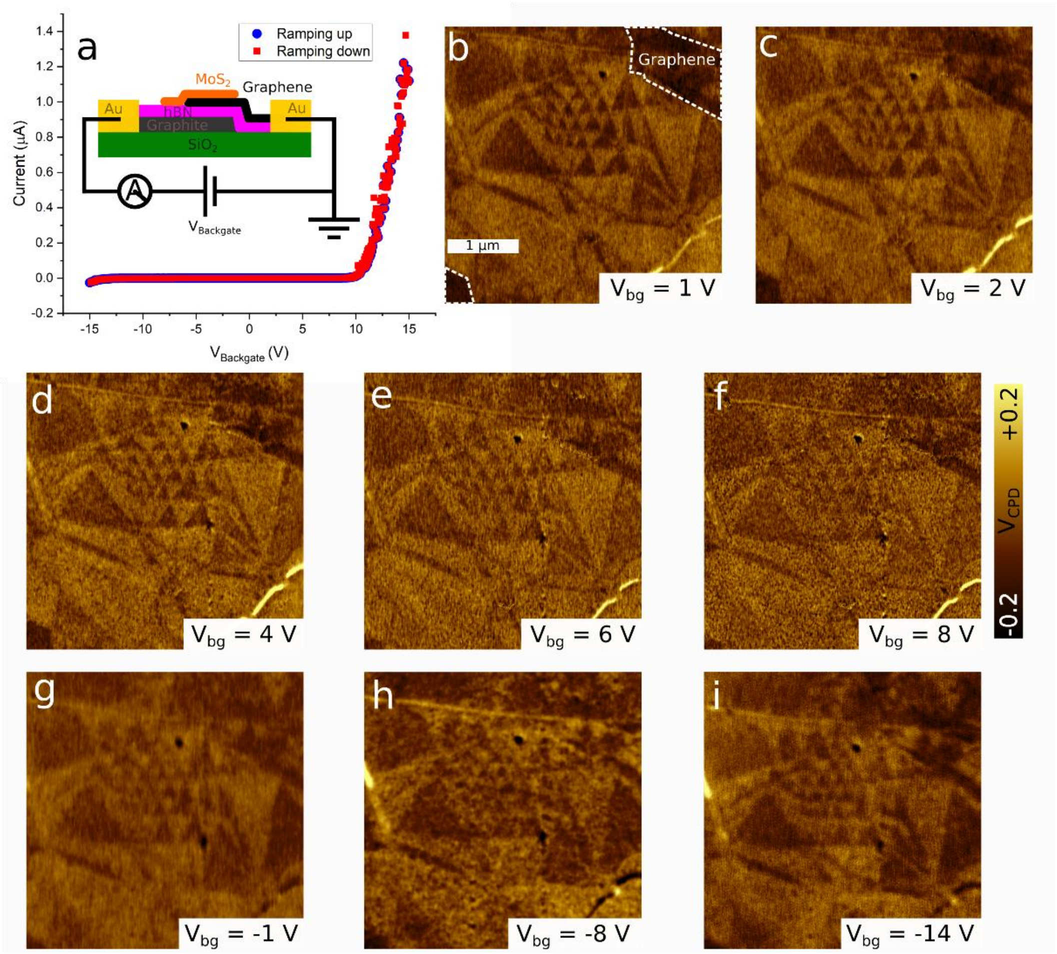

Figure S9: Effect of electrostatic doping. (a) Current as a function of applied back gate voltage blue/red sweeping voltage up/down. Schematic shows the device structure and electrical connections. **(b)** to **(f)** KPFM images at different applied back gate voltages from 1 V **(b)** to 8 V **(f)**.

Section 6: Contact mode AFM switching of the domains

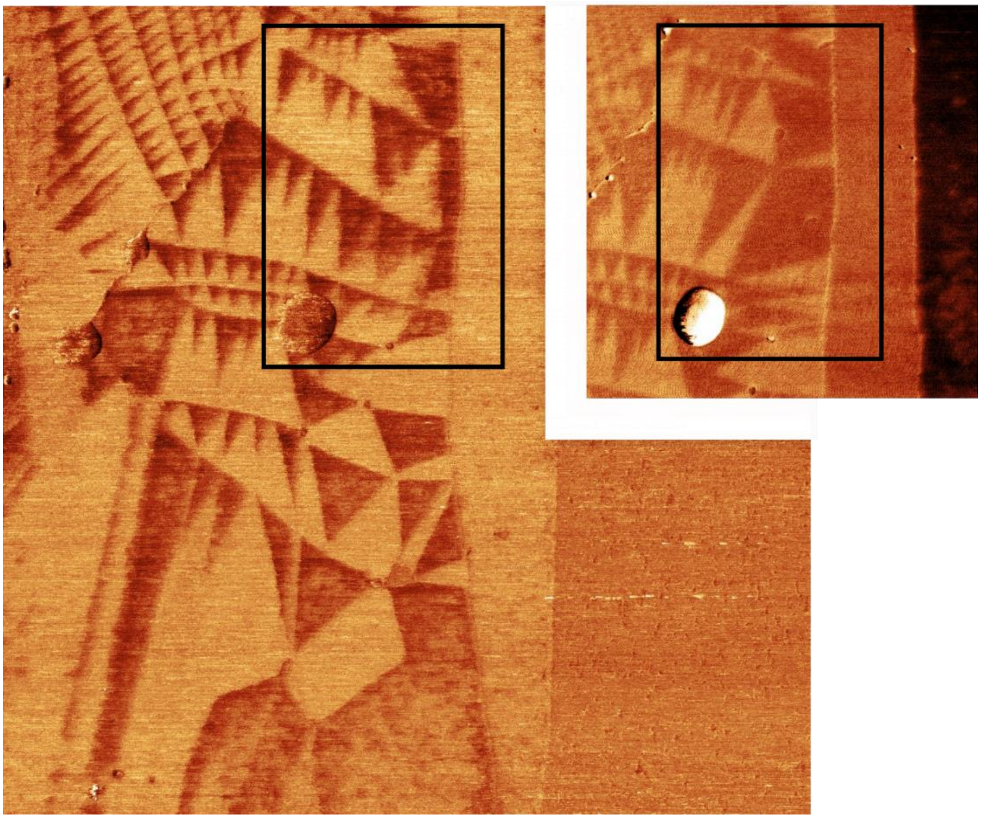

Figure S10: Mechanical switching of the 3R domains. Main panel: EFM mapping [16] of TMD domains acquired on twisted bilayer MoS_2 placed on graphite substrate. Boxed area was subsequently scanned in contact mode with no bias applied. **Inset:** Resulting EFM map showing significant rearrangement of the of the domain structure caused by mechanical action of the tip.

Electrostatic force microscopy (EFM) was performed on samples of twisted bilayer 3R-type MoS_2 placed on an exfoliated graphite crystal. The main phase contrast (second pass) domain map was aqcuired at zero bias voltage with a lift height of 13 nm. The inset phase image was acquired at zero bias voltage and a second pass lift height of 13 nm (also in EFM mode). The second image was aquired after -2V was applied (to the region in outlined in black) in piezoresonse force microsocpy scan of the boxed area. Here, the applied AC drive amplitude was 10 V and the same area was scanned thrree times before re-imaging using EFM. The high bias voltage and mechanical forces applied to the sample during PFM resulted in clear domain redistribution. Both imaging modes were performed on a Bruker Icon AFM using a Pt/Ir metal-coated tip (budget sensors ElectriMulti75-G).

Section 7: Electronic transport measurements

Our devices for electrical measurements were made from few-layer MoS_2 with different stacking configurations and encapsulated in hBN. They were shaped into the standard Hall bar geometry using electron beam lithography followed by reactive ion etching. We used the standard procedures to selectively etch the top hBN layer and deposit 5 nm Ti / 100 nm Au contacts to MoS_2. The top gate served a self-aligned etch mask and only affected the main channel, whereas the bottom gate (Si wafer) was global, inducing carriers in both main channel and contact leads. At zero gate voltages, all the devices were found insulating, and it required an applied electric field of ~0.1 V nm^{-1} or, equivalently, electron density of $\sim 2\times 10^{12}$ cm^{-2} to start inducing mobile carriers. Therefore, to study the devices' conductivity G, we chose to sweep the top gate voltage V_t while keeping a finite positive V_b, which was essential to induce electrical conductance in the contact regions. Because charge carrier mobility in few-layer MoS_2 at room temperature is only of the order of ~1 cm^2 V^{-1} s^{-1}, our devices exhibited high resistivity (often above 1 MΩ) and, therefore, only two-probe DC measurements were possible in most cases. However, if resistivity was low enough (e.g., trilayer devices at high electron doping) we have also used four-probe DC measurements to ensure consistency with the two-probe results. This approach combined with the use of various two-probe configurations proved that the measured conductivity was dominated by the main channel. Most importantly, the reported conductivity and its hysteresis were recorded as a function of top gate voltage, which inherently avoided any contribution from contact regions and leads, that is, outside the main MoS_2 channel (see the micrograph in Fig. S11). Because of high resistivity of few-layer MoS_2 (often above 1 MΩ), only two-probe DC measurements were possible. We focus below on the data obtained for devices made from two marginally twisted trilayers (zero θ) and two bilayers twisted by 60 or 180°. These stacking configurations are expected to result in an interface with ferroelectric domains as discussed above. Although we also made zero-twisted-monolayer devices, their contact resistances were too high (> 1GΩ) to reliably detect hysteresis. As a reference, we used few-layer MoS_2 with the standard 2H stacking.

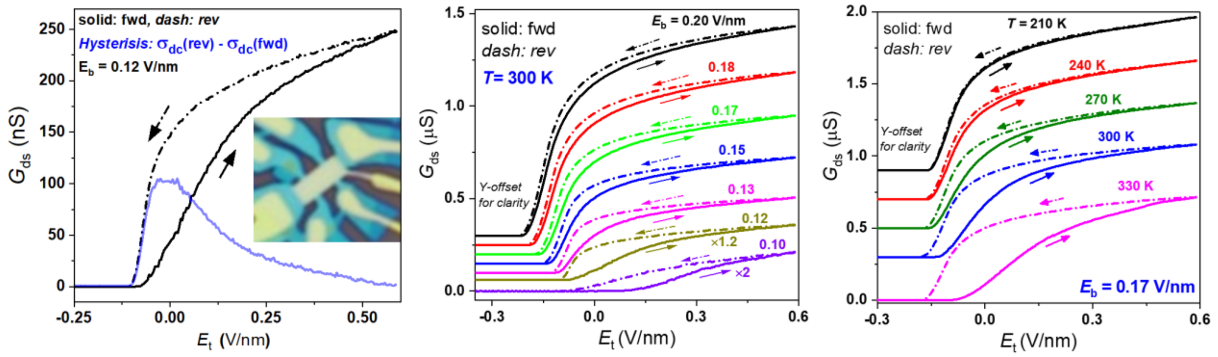

Figure S11. Hysteresis in electron transport for marginally twisted MoS_2. (A-C) Hysteresis in drain-source conductance (G_{ds}) as a function of top-gate electric field (E_t) as observed in two-terminal DC measurements. (A) An example of typical hysteresis loops for a given back-gate electric field E_b = 0.12 V/nm. The blue curve is the difference in conductance between reverse and forward sweep directions. Inset: Optical micrograph of one of our devices; in this case, 3L MoS_2 on top of another 3L MoS_2 with nominally zero twist angle. B) Same as in (A) for different E_b. (C) Hysteretic loops notably change with temperature indicating that the domain structure becomes increasingly less mobile at low T. All the G_{ds} curves were obtained by sweeping E_t from -0.58 to +0.58 V/nm and then back. We used the to gate for recording hysteresis because it covers only the twisted region whereas the bottom gate influences a much larger area, including contact regions.

The experimental curves $G(E_t, E_b)$ for reference devices were non-hysteretic as expected for 2H MoS_2 (Fig. S13). In contrast, pronounced hysteresis in electrical response was observed for marginally twisted devices, if we swept gate voltages up and down. This behaviour manifested in both zero-twisted double trilayers and monolayers. The recorded hysteresis loops show that, in the first approximation, they can be viewed as a shift of $G(E_t)$ curves for sweeps up and down (see Fig. 4). The shift and its direction are consistent with a remnant electric field that is built-in at the interface and acts against the applied field for sweeps up but adds to it for sweeps down. At zero V_t, the built-in field was sufficient to make the channel considerably either more or less conductive, depending on whether positive or negative gate voltage was previously applied to the devices, respectively. The difference between the resulting G depended on amplitude and time the ferroelectric interface was exposed to the applied electric field (Supplementary Fig. S12). The larger the applied E_t and the longer it was applied, the larger was the difference in G for the two resulting states. The ferroelectricity was characterised by long relaxation times, of the order of 10 s at room T, which rapidly increased with decreasing temperature. For a constant sweep rates (typically, 1 V per min), this resulted in hysteresis loops getting larger above room T and shrinking below it. This behaviour indicates that ferroelectric domain walls could creep slowly into their final positions. Interestingly, similar devices but made from few-layer MoS_2 with 3R stacking, which is known to be an intrinsic ferroelectric, were found impossible to switch even by the highest accessible $E \gtrsim 0.5$ V/nm, limited by the gate dielectric breakdown (Fig. S13). This suggests that domain walls at the artificially-made ferroelectric interfaces of the twisted

MoS_2 layers experienced considerably lower barriers for their movement than the generation of domain walls at edges of fully polarized 3R devices.

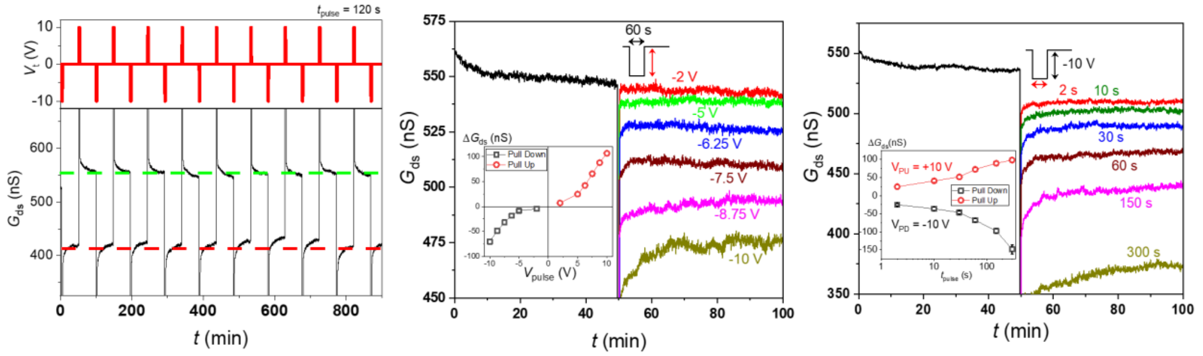

Figure S12. Changes in ferroelectric polarization of the device in Fig. 3 of the main text using voltage pulses of different amplitude and duration. For all the shown curves E_b was fixed at 0.17 V/nm. (A) Voltage pulses with amplitude V_{pulse} = ± 10 V (E_t = ± 0.58 V/nm) and duration of 120 s were applied to the top gate as shown in the top panel. G_{ds} reproducibly switched between two states that had higher and lower conductance after positive and negative pulses, respectively (bottom panel). The states are indicated by the red and green curves and differ by ~150 nS in conductance. (B) Temporal response $G_{ds}(t)$ using the same pulse duration of 60 s but varying amplitude V_{pulse}. The initial state for all the curves was achieved as shown in (A) using pulses of +10 V and waiting for 10 mins. Then the recording started (black curve). After further 50 mins a negative pulse was applied. The final states are color-coded. Inset: changes in conductance for pulses of different amplitudes and polarities. (C) G_{ds} for pulses of the same amplitude (-10 V) but of different duration (color-coded). The initial (black) state is the same as in (B). Inset: Resulting changes in conductance as a function of pulse duration.

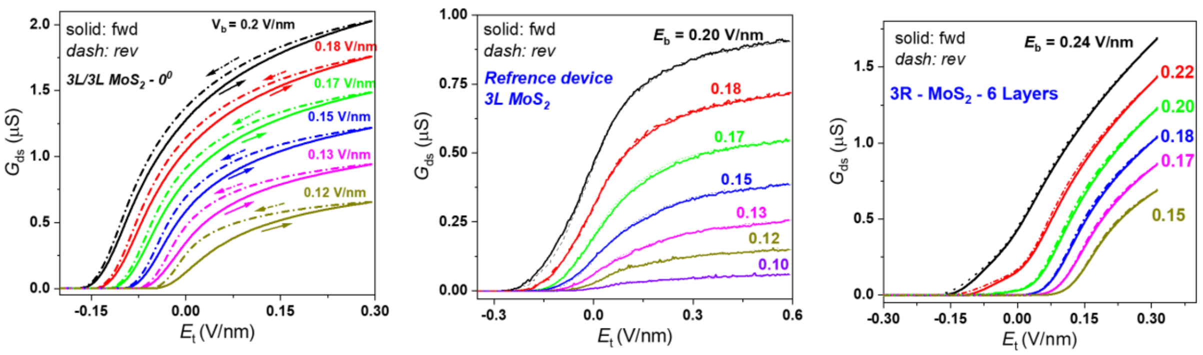

Figure S13. Comparison with reference devices. Measurements as in Fig. 3b of the main text (A) Another artificial ferroelectric obtained by stacking two MoS_2 trilayers on top of each other at zero twist angle. (B) Non-hysteretic response of a reference device made from trilayer MoS_2 with standard ABA stacking. (C) Same as in B but for 3R-MoS_2 (ferroelectric polymorph with ABC stacking). Total number of layers is six (ABCABC). The tiny differences between sweeps up and down in (B, C) are due to time delays and are left deliberately to indicate that the presence of sweeps in both directions.

Section 8: Modelling of the ferroelectric interlayer charge transfer in 3R MoS_2 bilayrs.

Ab initio Density Functional Theory (DFT) calculations have been carried out as in Ref. [17], [18], using the package Quantum ESPRESSO [19]. The exchange-correlation functional is approximated using the generalized gradient approximation (GGA) in the scheme of Perdew-Burke-Ernzerhof (PBE) [20]. A plane-wave cut-off of 80 Ry and a Monkhorst-Pack [21] grid of 12x12x1 k-points have been used for all calculations. Spin-orbit coupling is taken into account by using full-relativisitic pseudopotentials. For all calculations, the double bilayer supercell method described in Ref. [18] has been used to avoid the mismatch of the vacuum potentials at the supercell boundary. The expression for the potential drop,

$$\Delta V^{FE}(\boldsymbol{r}_0, d-d_0) = \Delta_a e^{-q(d-d_0)} \sum_{j=1,2,3} \sin(\boldsymbol{G}_j \cdot \boldsymbol{r}_0), \qquad \text{(S1)}$$

has been parametrized using DFT calculations for a range of in-plane offset between the layers, $\boldsymbol{r}_0$, and interlayer distances, d, as in Ref. [15]. Here, $\boldsymbol{G}_j$ are the shortest reciprocal vectors of TMD monolayers, $\boldsymbol{r}_0$ is the in-plane offset between the layers, and $d - d_0$ is the interlayer distance, counted from d_0=6.36 Å (for details, see in Refs [17], [18], [22]), $\Delta_a = 16$ mV, and $q = 2.215$ Å^{-1}, which were determined from DFT computed bilayer Hartree potentials, fitted using Eq. (S1). For 3R stacking, with $d - d_0 = -0.21$ Å, we find $\Delta^p = 66$ mV.

Then, we estimate that areal density of ferroelectric dipole moment locally for each offset $\boldsymbol{r}_0$ is

$$P(\boldsymbol{r}_0) = \epsilon_0 \Delta V^{FE},$$

For 3R domains, this agrees with the value, $P = \pm 3.8 \times 10^{-3}\, e$/nm, determined by a direct integration of the DFT-computed FE charge density, quoted in the main text. For ploting the maps of P and Δ^p in Figs 2 and 3, we obtained the in-plane deformations $\boldsymbol{u}^{t/b}$ in the top/bottom layer of the recostructure bilayer using the multiscale approach of Ref. [22] (see Section S8 for details), and, then, substituted local offset, $\boldsymbol{r}_0 = \theta\hat{\boldsymbol{z}} \times \boldsymbol{r} + \boldsymbol{u}^t - \boldsymbol{u}^b$, into Eq. (S1), where θ is the twist angle related to the domain size as $\ell = a/\theta$, and a is lattice constant of MoS_2.

Section 9 Quantitative analysis of the domain wall network.

Below, we describe in details the model used to analyse the domain wall network in twisted MoS_2 bilayers placed in out-of-plane displacement field D. This model employs an energy functional which accounts for the orientation-dependence of the energy of partial dislocations, $\overline{w} + \widetilde{w}\sin^2\phi$, where ϕ is an angle between the dislocation axis and armchair direction in MoS_2 crystal, which was found earlier to be energetically most favourable, see Fig. S14a. The values of parameters, $\overline{w} = 1.05$ eV/nm

and $\widetilde{w} = 0.68$ eV/nm were determined using the data in Ref.[22]. We also take into account a possibility that two partial discolorations merge into a full screw dislocation, with the energetically preferable orientation along zigzag axis in the crystal, for which its energy per unit length is u=2.24 eV/nm. We also take into account for the difference of electrostatic energies in oppositely polarised ferroelectric Mo^tS^b and Mo^bS^t domains, $\pm\frac{D\,P}{\chi\epsilon_0}$. The FSD energy was obtained from the lattice relaxation analysis, similar to that performed in Ref.[22]. For each partial dislocation in the domain network with a period ℓ we introduce a function $y(x)$ ($\delta < x < \ell - \delta$) that describes its transversal displacements from a straight line connecting the network nodes. The offset δ accounts for a possible formation of full screw dislocation segments (see Fig. S14b). To find the exact form of partial dislocations, as a function of the out-of-plane displacement field, we minimise energy,

$$\mathcal{E}_\ell\,[y(x)] = 3\int_\delta^{\ell-\delta}\left[\left(\bar{w} + \widetilde{w}\frac{y'^2}{1+y'^2}\right)\sqrt{1+y'^2} - 2\frac{D\,P}{\chi\epsilon_0}y\right]dx + 2\sqrt{3}\left[u - \frac{D\,P}{\chi\epsilon_0}\delta\right]\delta\,. \quad \text{(S2)}$$

Here, the first term in the integral (with a factor $\sqrt{1+y'^2}$) describes energy of stretching PD and its dependence on the deviation of the PD axis from the closest armchair direction in the crystal (factor $\frac{y'^2}{1+y'^2}$). The second term stands for the energy gain from increasing the area of domain with the stacking promoted by the electric field. The formation of a full dislocation – due to merging of two partial dislocations – is expected to develop from the network nodes (intervals $0 < x < \delta$; $\ell - \delta < x < \ell$), and its influence on the total energy is describes by the last term in $\mathcal{E}_\ell\,[y(x)]$. Using the variational principle, we arrive at an equation,

$$y'' = -\frac{2DP}{\chi\epsilon_0}\frac{\left(1+y'^2\right)^{\frac{5}{2}}}{(\bar{w}+2\widetilde{w})+(\bar{w}-\widetilde{w})y'^2}\,, \quad \text{(S3)}$$

for the shape of the PD part of the domain wall.

For a small displacement field, the DW deformations are weak, $y'^2 \ll 1$, so that the functional in equation (S2) can be reduced to the Eq. (1) of the main text, and solution of Eq. (S3) can be approximated by a parabola,

$$y(x) \approx \frac{\frac{DP}{\chi\epsilon_0}}{\bar{w}+2\widetilde{w}}x(\ell - x). \quad \text{(S4)}$$

In Fig. S14c, we compare this approximate solution to the one obtained numerically from Eq. (S3), finding a close agreement. Therefore, we use the approximate analytical form in Eq. (S4) to analyse

the evolution of shapes of smaller domains in Fig. 2 and to fit the value of the coupling parameter, $\chi \approx 1.5$, using the experimental data.

For displacement fields at a threshold, D_*, pairs or PDs merge together (that is, they touch each other: $\phi°(0) = -\phi°(\ell) = 30°$, which is equivalent to $y'(0) = -y'(\ell) = 1/\sqrt{3}$) near the domain network nodes ($x = 0$; $x = \ell$). Integrating Eq. (1) once and using the above conditions, we find that the threshold displacement field scales as $D_* \propto \ell^{-1}$ with the domain size, and, using the coupling parameter fit to the experimental data below the threshold, we find $\epsilon_0^{-1} D_* = \frac{\chi\left(\frac{\overline{w}}{2}+\frac{7\widetilde{w}}{8}\right)}{P\ell} \approx 440\ V/\ell$ (which the value quoted in the main text). To mention, if we use an approximate parabolic solution, we would get an overestimated value for threshold, $\epsilon_0^{-1}\widetilde{D}_* \approx 500\ V/\ell$, so that everywhere below and also in the main text we use the exactly evaluated threshold value.

Above that threshold, the DW is composed of two parts: (a) a perfect screw dislocation (PSD segment with a length $\frac{\delta}{\sqrt{3}}$) oriented along a zigzag direction, separating pairs of expanded energetically favourable 3D domains near each domain network node, and (b) segments of PDs splitting from these PSDs and touching each other at the splitting points at $x = \delta$, and $x = \ell - \delta$, with $y'(\delta) = -y'(\ell - \delta) = 1/\sqrt{3}$. The latter appear to have a universal shape, scalable by a field-dependent factor. To demonstrate such scalability, we substitute $x = \ell\tilde{x}\frac{D_*}{D} + \delta$, $y = \ell\tilde{y}\frac{D_*}{D} + \frac{\delta}{\sqrt{3}}$ into Eq. (S3), leading to the rescaled ordinary differential equation (same for all displacement fields),

$$\tilde{y}'' = -\frac{\overline{w}+\frac{7}{4}\widetilde{w}}{\overline{w}+2\widetilde{w}} \frac{\left(1+\tilde{y}'^2\right)^{\frac{5}{2}}}{1+\frac{\overline{w}-\widetilde{w}}{\overline{w}+2\widetilde{w}}\tilde{y}'^2}, \quad 0 \le \tilde{x} \le 1; \;\; \tilde{y}'^{(0)} = -\tilde{y}'(1) = 1/\sqrt{3}. \qquad \text{(S5)}$$

To find the universal solution we integrate (S5) across $0 \le \tilde{x} \le \frac{1}{2}$ with a boundary condition $\tilde{y}'\left(\frac{1}{2}\right) = 0$ (and then use a symmetrically reflected solution for the rest of the interval), resulting in

$$\tilde{y}(\tilde{x}) = \int_0^{\tilde{x}} \frac{f(\tilde{x}')}{\sqrt{1-f^2(\tilde{x}')}} d\tilde{x}', \qquad \text{(S6)}$$

where $f(\tilde{x})$ is the real root of a cubic polynomial,

$$f^3 - \left(\frac{\overline{w}}{\widetilde{w}} + 2\right) f - \left(\frac{\overline{w}}{\widetilde{w}} + \frac{7}{4}\right)\left(\tilde{x} - \frac{1}{2}\right) = 0.$$

In Fig. S14c, we compare the resulting solution (blue lines) with an approximate parabolic form of PDs obtained for the quadratic expansion used in Eq. (1) in the main text (red lines),

$$y(x) = \frac{\frac{DP}{\chi\epsilon_0}}{\bar{w}+2\tilde{w}}(x-\delta)(\ell-\delta-x)+\frac{\delta}{\sqrt{3}}, \quad \delta < x < \ell-\delta, \qquad \text{(S7)}$$

matching it with the FSD ends at $\delta = \frac{\ell}{2}\left(1-\frac{D_*}{D}\right)$. We find these two are sufficiently close to each other for the entire studied displacement field range to justify the use of parabolic shape of domain walls. Moreover, in the panels of Fig. S14d we compare the approximate 'parabolic' solutions for the form of the domain wall network with the results of the mesoscale lattice relaxation (see in section S10 below), also, with a good agreement between those results.

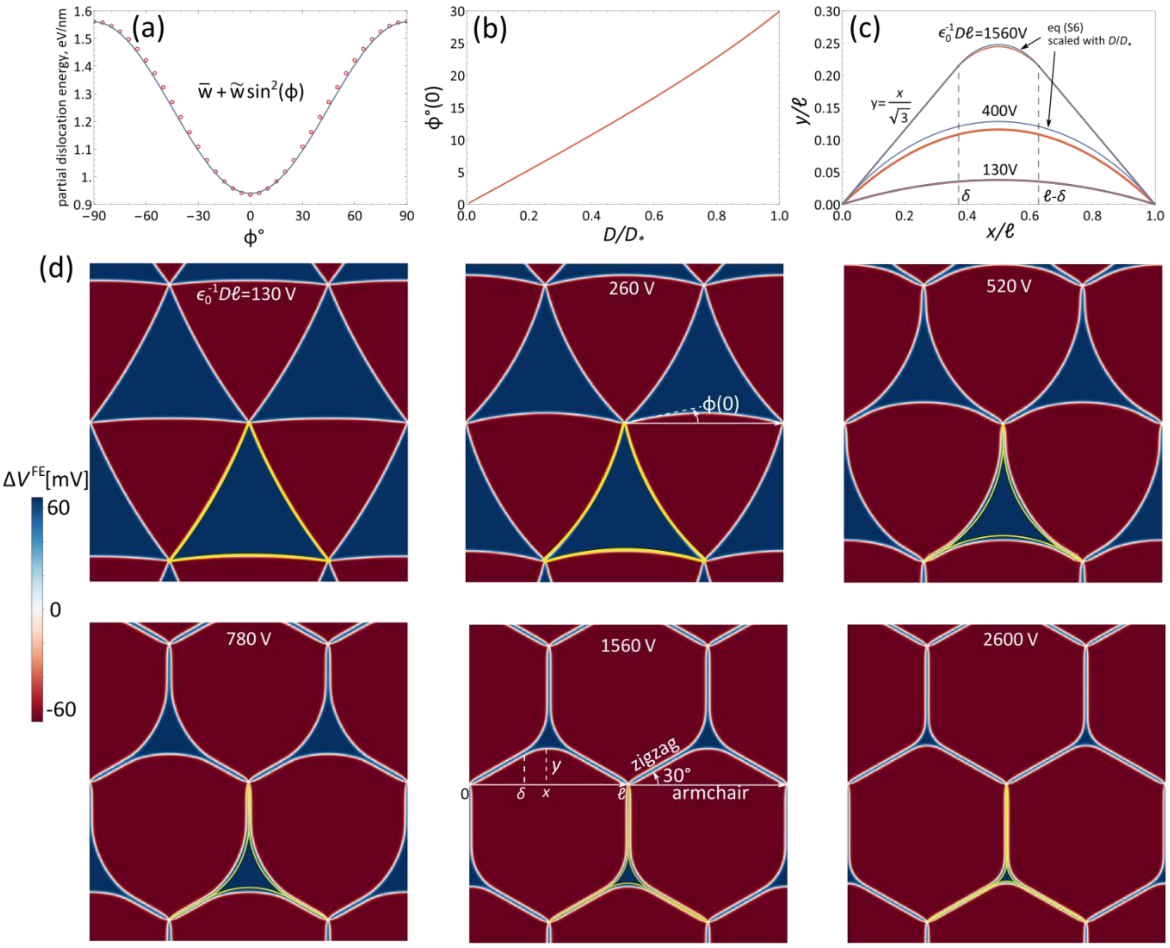

Figure S14. (a) PD energy computed in Ref.[22] for various orientations of DW with respect to the energetically most favourable armchair direction (red circles), described using analytical expression used in Eqs. (1) and (S2), $\bar{w}+\tilde{w}\sin^2\phi$ (blue lines). (b) The computed deviation or the PD axis from the armchair direction near the domain network nodes as a function of displacement field, reaching 30° (zigzag direction in MoS_2 crystal) at that the threshold value, $\epsilon_0^{-1}D_* = 440\ V/\ell$. (c) Comparison of approximate and exact shapes for the domain wall in various displacement fields, as described in the text. (d) The calculated domain network evolution (from Eqs. (2) and (S7)) superimposed over the map of potential created by the ferroelectric double-layer at the MoS_2/MoS_2 interface with stacking computed upon the mesoscale lattice relaxation, as described in sections and S9.

Section 10 Mesoscale lattice reconstruction and mapping potential profiles in twisted bilayers

To calculate lattice reconstruction in across moiré pattern in twisted MoS_2 bilayers, we use the earlier-developed multiscale modelling approach [2], [22], [23]. Within this approach lattice relaxation is described by local atomic displacements $\boldsymbol{u}^{(t/b)}$ in top/bottom monolayers. To find the displacements, we minimise the total energy of the bilayer,

$$\mathcal{E}_{2D} = \int d^2\boldsymbol{r}\left\{\sum_{a=t,b}\left[\frac{1}{2}\lambda\left(u_{ii}^{(a)}\right)^{\mathbf{2}} + \mu u_{ij}^{(a)}u_{ji}^{(a)}\right] + W_{adh}\left(\boldsymbol{r_0}(\boldsymbol{r})\right) + \chi^{-1}D\Delta V^{FE}\left(\boldsymbol{r_0}(\boldsymbol{r})\right)\right\}. \quad \text{(S8)}$$

The first term in curly brackets is responsible for elastic energy of top and bottom monolayers characterised by strain tensor $u_{ij}^{(t/b)} = \frac{\partial_i u_j^{(t/b)} + \partial_j u_i^{(t/b)}}{2}$ and elastic moduli λ, μ of MoS_2 monolayer. The second term describes adhesion energy between top and bottom layers,

$$W_{adh}(\boldsymbol{r}_0) = -\kappa Z^2(\boldsymbol{r}_0) + \sum_{n=1,2,3}(w_1 + w_2)\cos \boldsymbol{G}_n \cdot \boldsymbol{r}_0\,, \quad \text{(S9)}$$

defined for local stacking, parameterised by the interlayer lattice offset, $\boldsymbol{r}_0(\boldsymbol{r}) = \theta\hat{\boldsymbol{z}} \times \boldsymbol{r} + \boldsymbol{u}^{(t)} - \boldsymbol{u}^{(b)}$, and accounting to the variation of the optimal offset-dependent local interlayer distance,

$$Z(\boldsymbol{r}_0) = \frac{1}{2\kappa}\sum_{n=1,2,3}(w_1 Q + w_2 G)\cos \boldsymbol{G}_n \boldsymbol{r}_0. \quad \text{(S10)}$$

Here, $G = |\boldsymbol{G}_{1,2,3}|$ where $\boldsymbol{G}_{1,2,3}$ are same three shortest reciprocal lattice vectors of MoS_2 monolayer as in Eq. (S1), related by 120°-rotation (for details, see Refs. [2], [17], [18], [22], [23]). In Table S1, we list the magnitudes of the elastic moduli and adhesion energy parameters, determined in the earlier ab initio simulations using density functional theory [17,15]. The last term in Eq. (S8) describes interaction of out-of-plane displacement field with the local polarization $P = \Delta V^{FE}(\boldsymbol{r}_0, d)$, induced by the interlayer charge transfer, as a function of position in the domain structure**.** The latter has the same spatial distribution as the double-charge layer potential and its coupling to the externally controlled displacement field, D, is parametrized by a dimensionless coupling constant, χ, which was determined ($\chi \approx 1.5$) from the comparison between the modelled and experimentally observed variation domain shapes (see in Section S9).

To find atomic displacements, which determine local stacking, we numerically solve a system of four Euler-Lagrange equations discretized on a sufficiently dense grid using interior point method implemented in GEKKO Optimization Suite package [24].

Table S1. Elastic moduli and parameters in adhesion energy used in the lattice relaxation calculation

λ, N/nm	μ, N/nm	κ, eV/nm^4	w_1, eV/nm^2	w_2, eV/nm^2	Q, nm^{-1}
83.2	70.9	214	0.1727	0.0186	30.53

The obtained numerical solution was used in Eq. (S1), taking into account the dependence of the interlayer charge transfer on the interlayer distance variation, $d - d_0 = Z(\boldsymbol{r}_0)$, and including all the obtained information on the local stacking $\boldsymbol{r}_0(\boldsymbol{r}) = \theta\hat{z} \times \boldsymbol{r} + \boldsymbol{u}^{(t)} - \boldsymbol{u}^{(b)}$. Then, using parameters listed in Table S1, we computed the maps shown in Fig S13d, Fig. 2g-I, and a detailed profile of the potential variation at the domain wall in the inset in Fig. 3, where we took into account piezoelectric charges created by MoS_2 deformations near the domain walls [18] (these charges have the oposite sign in the two monolayer, and they partly smothen the potential profiles at the legth scale of the domain wall width).

[1] K. Kim *et al.*, “Van der Waals Heterostructures with High Accuracy Rotational Alignment,” *Nano Lett.*, vol. 16, no. 3, pp. 1989–1995, 2016.

[2] A. Weston *et al.*, “Atomic reconstruction in twisted bilayers of transition metal dichalcogenides,” *Nat. Nanotechnol.*, vol. 15, no. 7, pp. 592–597, Nov. 2020.

[3] R. F. Egerton, P. Li, and M. Malac, “Radiation damage in the TEM and SEM,” in *Micron*, 2004, vol. 35, no. 6, pp. 399–409.

[4] M. R. Rosenberger, H.-J. Chuang, K. M. McCreary, A. T. Hanbicki, S. V. Sivaram, and B. T. Jonker, “Nano-‘Squeegee’ for the Creation of Clean 2D Material Interfaces,” *ACS Appl. Mater. Interfaces*, vol. 10, no. 12, pp. 10379–10387, Mar. 2018.

[5] “NT-MDT Application note: SINGLE-PASS MEASUREMENTS IN ATOMIC FORCE MICROSCOPY: KELVIN PROBE FORCE MICROSCOPY AND LOCAL DIELECTRIC STUDIES.” .

[6] T. Glatzel, S. Sadewasser, and Mc. Lux-Steiner, “Amplitude or frequency modulation-detection in Kelvin probe force microscopy,” *Appl. Surf. Sci.*, vol. 210, no. 1–2, pp. 84–89, Mar. 2003.

[7] G. Cohen *et al.*, “Reconstruction of surface potential from Kelvin probe force microscopy images,” *Nanotechnology*, vol. 24, no. 29, p. 295702, Jul. 2013.

[8] U. Zerweck, C. Loppacher, T. Otto, S. Grafström, and L. M. Eng, “Accuracy and resolution limits of Kelvin probe force microscopy,” *Phys. Rev. B*, vol. 71, no. 12, p. 125424, Mar. 2005.

[9] E. G. Castanon *et al.*, “Calibrated kelvin-probe force microscopy of 2d materials using pt-coated probes,” *J. Phys. Commun.*, vol. 4, no. 9, pp. 1–13, Sep. 2020.

[10] J. Xu, G. Bai, J. Li, and W. Li, “Inhomogeneous probe surface induced effect in Kelvin probe force microscopy,” *J. Appl. Phys.*, vol. 127, no. 18, p. 184302, May 2020.

[11] C. Örnek, C. Leygraf, and J. Pan, “On the Volta potential measured by SKPFM–fundamental and practical aspects with relevance to corrosion science,” *Corros. Eng. Sci. Technol.*, vol. 54,

no. 3, pp. 185–198, 2019.

[12] V. Panchal, R. Pearce, R. Yakimova, A. Tzalenchuk, and O. Kazakova, “Standardization of surface potential measurements of graphene domains,” *Sci. Rep.*, vol. 3, no. 1, pp. 1–8, Sep. 2013.

[13] C. Melios, V. Panchal, C. E. Giusca, W. Strupiński, S. R. P. Silva, and O. Kazakova, “Carrier type inversion in quasi-free standing graphene: Studies of local electronic and structural properties,” *Sci. Rep.*, vol. 5, no. 1, pp. 1–8, Jun. 2015.

[14] S. Barbet, M. Popoff, H. Diesinger, D. Deresmes, D. Théron, and T. Mélin, “Cross-talk artefacts in Kelvin probe force microscopy imaging: A comprehensive study,” *J. Appl. Phys.*, vol. 115, no. 14, p. 144313, Apr. 2014.

[15] Y. Wu and M. A. Shannon, “ac driving amplitude dependent systematic error in scanning Kelvin probe microscope measurements: Detection and correction,” *Rev. Sci. Instrum.*, vol. 77, no. 4, p. 043711, Apr. 2006.

[16] C. R. Woods *et al.*, “Charge-polarized interfacial superlattices in marginally twisted hexagonal boron nitride,” *Nat. Commun.*, vol. 12, no. 1, pp. 1–7, Dec. 2021.

[17] F. Ferreira, S. J. Magorrian, V. V Enaldiev, D. A. Ruiz-Tijerina, and V. I. Fal’ko, “Band energy landscapes in twisted homobilayers of transition metal dichalcogenides,” *Appl. Phys. Lett.*, vol. 118, no. 24, 2021.

[18] F. Ferreira, V. V. Enaldiev, V. I. Fal’ko, S. J. Magorrian, V. I. Fal’ko, and S. J. Magorrian, “Weak ferroelectric charge transfer in layer-asymmetric bilayers of 2D semiconductors,” *Sci. Rep.*, vol. 11, no. 1, pp. 1–10, Mar. 2021.

[19] P. Giannozzi *et al.*, “Quantum ESPRESSO toward the exascale,” *J. Chem. Phys.*, vol. 152, no. 15, p. 154105, Apr. 2020.

[20] J. P. Perdew, K. Burke, and M. Ernzerhof, “Generalized Gradient Approximation Made Simple,” *Phys. Rev. Lett.*, vol. 77, no. 18, pp. 3865–3868, Oct. 1996.

[21] H. J. Monkhorst and J. D. Pack, “Special points for Brillouin-zone integrations,” *Phys. Rev. B*, vol. 13, no. 12, pp. 5188–5192, Jun. 1976.

[22] V. V. Enaldiev, V. Zólyomi, C. Yelgel, S. J. Magorrian, and V. I. Fal’ko, “Stacking Domains and Dislocation Networks in Marginally Twisted Bilayers of Transition Metal Dichalcogenides,” *Phys. Rev. Lett.*, vol. 124, no. 20, p. 206101, Nov. 2020.

[23] V. V Enaldiev, F. Ferreira, S. J. Magorrian, and V. I. Fal’ko, “Piezoelectric networks and ferroelectric domains in twistronic superlattices in WS2/MoS2 and WSe2/MoSe2 bilayers,” *2D Mater.*, vol. 8, no. 2, p. 025030, Feb. 2021.

[24] L. D. R. Beal, D. C. Hill, R. A. Martin, and J. D. Hedengren, “GEKKO Optimization Suite,” *Process. 2018, Vol. 6, Page 106*, vol. 6, no. 8, p. 106, Jul. 2018.
